# Fear in dreams and in wakefulness: Evidence for day/night affective homeostasis

**DOI:** 10.1002/hbm.24843

**Published:** 2019-10-30

**Authors:** Virginie Sterpenich, Lampros Perogamvros, Giulio Tononi, Sophie Schwartz

**Affiliations:** ^1^ Department of Basic Neurosciences, Faculty of Medicine University of Geneva Geneva Switzerland; ^2^ Swiss Center for Affective Sciences University of Geneva Geneva Switzerland; ^3^ Center for Sleep Medicine, Division of Pulmonology, Department of Medicine Geneva University Hospitals Geneva Switzerland; ^4^ Wisconsin Institute for Sleep and Consciousness, Department of Psychiatry University of Wisconsin – Madison Madison Wisconsin

**Keywords:** dreaming, EEG, emotion regulation, fear, fMRI, insula, wakefulness

## Abstract

Recent neuroscientific theories have proposed that emotions experienced in dreams contribute to the resolution of emotional distress and preparation for future affective reactions. We addressed one emerging prediction, namely that experiencing fear in dreams is associated with more adapted responses to threatening signals during wakefulness. Using a stepwise approach across two studies, we identified brain regions activated when experiencing fear in dreams and showed that frightening dreams modulated the response of these same regions to threatening stimuli during wakefulness. Specifically, in Study 1, we performed serial awakenings in 18 participants recorded throughout the night with high‐density electroencephalography (EEG) and asked them whether they experienced any fear in their dreams. Insula and midcingulate cortex activity increased for dreams containing fear. In Study 2, we tested 89 participants and found that those reporting higher incidence of fear in their dreams showed reduced emotional arousal and fMRI response to fear‐eliciting stimuli in the insula, amygdala and midcingulate cortex, while awake. Consistent with better emotion regulation processes, the same participants displayed increased medial prefrontal cortex activity. These findings support that emotions in dreams and wakefulness engage similar neural substrates, and substantiate a link between emotional processes occurring during sleep and emotional brain functions during wakefulness.

## 1 INTRODUCTION

Converging evidence from human and animal research suggests functional links between sleep and emotional processes (Boyce, Glasgow, Williams, & Adamantidis, 2016; Perogamvros & Schwartz, 2012; Wagner, Hallschmid, Rasch, & Born, 2006; Walker & van der Helm, 2009). Chronic sleep disruption can lead to increased aggressiveness (Kamphuis, Meerlo, Koolhaas, & Lancel, 2012) and negative mood states (Zohar, Tzischinsky, Epstein, & Lavie, 2005), whereas affective disorders such as depression and post‐traumatic stress disorder (PTSD) are frequently associated with sleep abnormalities (e.g., insomnia and nightmares). Experimental evidence indicates that acute sleep deprivation impairs the prefrontal control over limbic regions during wakefulness, hence, exacerbating emotional responses to negative stimuli (Yoo, Gujar, Hu, Jolesz, & Walker, 2007). Neuroimaging and intracranial data further established that, during human sleep, emotional limbic networks are activated (e.g., Braun et al., 1997; Corsi‐Cabrera et al., 2016; Maquet et al., 1996; Nofzinger, Mintun, Wiseman, Kupfer, & Moore, 1997; Schabus et al., 2007). Together these findings indicate that sleep physiology may offer a permissive condition for affective information to be reprocessed and reorganized. Yet, it remains unsettled whether such emotion regulation processes also happen at the subjective, experiential level during sleep, and may be expressed in dreams. Several influential theoretical models formalized this idea. For example, the threat simulation theory postulated that dreaming may fulfill a neurobiological function by allowing an offline simulation of threatening events and rehearsal of threat‐avoidance skills, through the activation of a fear‐related amygdalocortical network (Revonsuo, 2000; Valli et al., 2005). Such mechanism would promote adapted behavioral responses in real life situations (Valli & Revonsuo, 2009). By contrast, other models suggested that dreaming would facilitate the resolution of current emotional conflict (Cartwright, Agargun, Kirkby, & Friedman, 2006; Cartwright, Luten, Young, Mercer, & Bears, 1998), the reduction of next‐day negative mood (Schredl, 2010) and extinction learning (Nielsen & Levin, 2007). Although these two main theoretical lines differ, because one focuses on the optimization of waking affective reactions (Perogamvros & Schwartz, 2012; Revonsuo, 2000) and the other on the resolution of current emotional distress (e.g., fear extinction; Nielsen & Levin, 2007), both converge to suggest that experiencing fear in dreams leads to more adapted responses to threatening signals during wakefulness (Scarpelli, Bartolacci, D'Atri, Gorgoni, & De Gennaro, 2019). The proposed mechanism is that memories from a person's affective history are replayed in the virtual and safe environment of the dream so that they can be reorganized (Nielsen & Levin, 2007; Perogamvros & Schwartz, 2012). From a neuroscience perspective, one key premise of these theoretical models is that experiencing emotions in dreams implicates the same brain circuits as in wakefulness (Hobson & Pace‐Schott, 2002; Schwartz, 2003). Preliminary evidence from two anatomical investigations showed that impaired structural integrity of the left amygdala was associated with reduced emotional intensity in dreams (Blake, Terburg, Balchin, van Honk, & Solms, 2019; De Gennaro et al., 2011).

Like during wakefulness, people experience a large variety of emotions in their dreams, with rapid eye movement (REM) dreaming being usually more emotionally loaded than non‐rapid eye movement (NREM) dreams (Carr & Nielsen, 2015; Smith et al., 2004). While some studies found a relative predominance of negative emotions, such as fear and anxiety, in dreams (Merritt, Stickgold, Pace‐Schott, Williams, & Hobson, 1994; Roussy et al., 2000), others reported a balance of positive and negative emotions (Schredl & Doll, 1998), or found that joy and emotions related to approach behaviors may prevail (Fosse, Stickgold, & Hobson, 2001; Malcolm‐Smith, Koopowitz, Pantelis, & Solms, 2012). When performing a lexicostatistical analysis of large data sets of dream reports, a clear dissociation between dreams containing basic, mostly fear‐related, emotions and those with other more social emotions (e.g., embarrassment, excitement, frustration) was found, highlighting distinct affective modes operating during dreaming, with fear in dreams representing a prevalent and biologically‐relevant emotional category (Revonsuo, 2000; Schwartz, 2004). Thus, if fear‐containing dreams serve an emotion regulation function, as hypothesized by the theoretical models, the stronger the recruitment of fear‐responsive brain regions (e.g., amygdala, cingulate cortex, and insula; see Phan, Wager, Taylor, & Liberzon, 2002) during dreaming, the weaker the response of these same regions to actual fear‐eliciting stimuli during wakefulness should be. This compensatory or homeostatic mechanism may also be accompanied by an enhanced recruitment of emotion regulation brain regions (such as the medial prefrontal cortex, mPFC, which is implicated in fear extinction) during wakefulness (Dunsmoor et al., 2019; Phelps, Delgado, Nearing, & LeDoux, 2004; Quirk, Likhtik, Pelletier, & Pare, 2003; Yoo et al., 2007).

Here, we collected dream reports and functional brain measures using high‐density EEG (hdEEG) and functional MRI (fMRI) across two studies to address the following questions: (a) do emotions in dreams (here fear‐related emotions) engage the same neural circuits as during wakefulness and (b) is there a link between emotions experienced in dreams and brain responses to emotional stimuli during wakefulness. By addressing these fundamental and complementary topics, we aim at clarifying the grounding conditions for the study of dreaming as pertaining to day/night affective homeostasis.

## 2 METHODS

### 2.1 Study 1: Neural correlates of fear in dreams

#### 2.1.1 Participants

Eighteen healthy participants were included in Study 1 (four males, age 39.77 ± 13.12 years, 25–63 [mean ± *SD*, range]). From these 18 participants, 12 (*N* = 12) were used for the analysis of fear versus no fear conditions in NREM sleep (N2 stage), while eight (*N* = 8) were used for the analysis of fear versus no fear conditions in REM sleep. All participants had no history of neurological or psychiatric disorder. Signed informed consent was obtained from all participants before the experiment, and ethical approval for the study was obtained from the University of Wisconsin‐Madison Institutional Review Board. This is a secondary analysis of previously acquired data, which were initially used in the article by Siclari et al. (2017).

### 2.2 Procedure

#### 2.2.1 Serial awakenings during sleep

**Figure 1:**
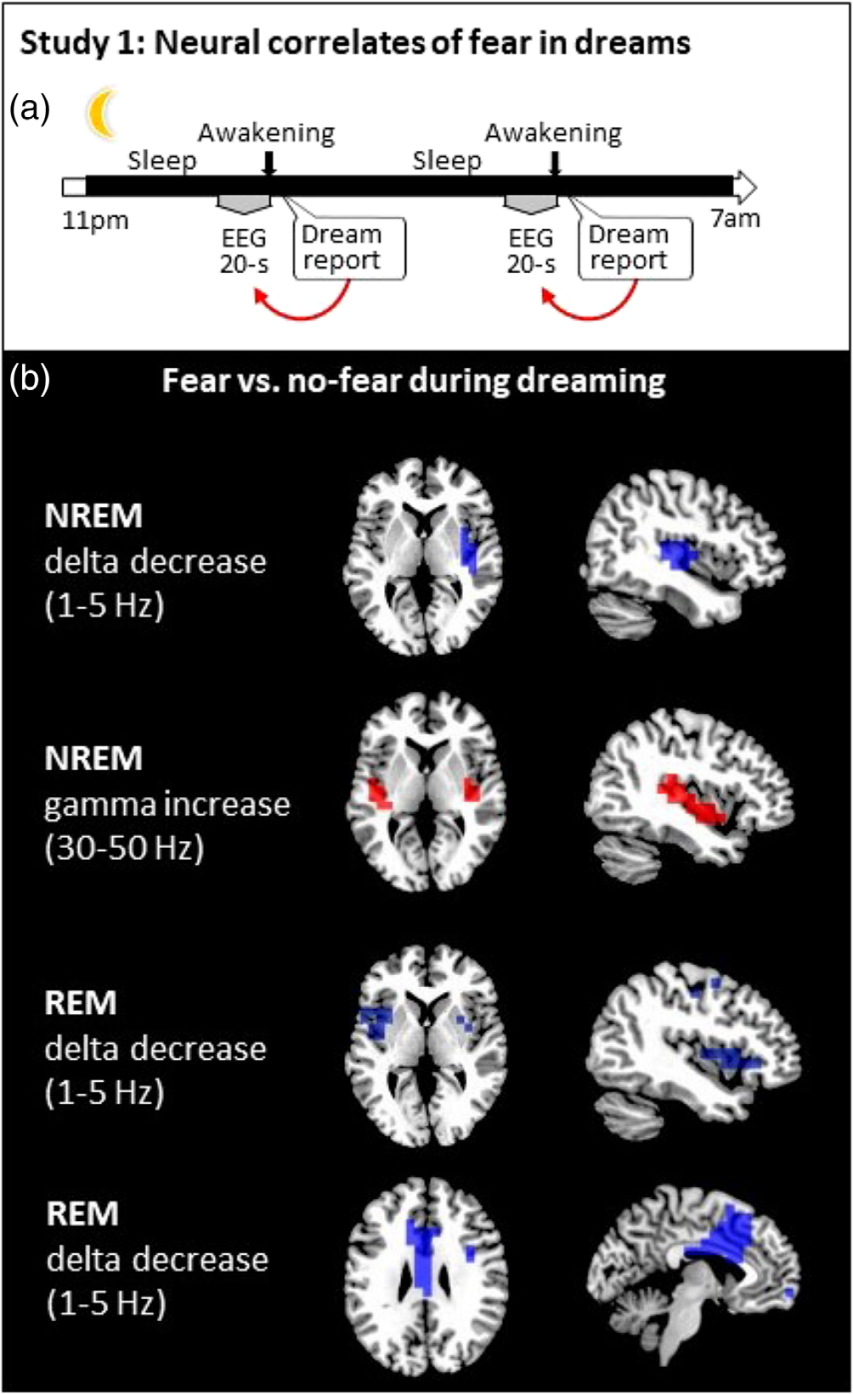
(a) Participants under hdEEG were awakened several times while sleeping in the sleep laboratory and were asked to report “the last thing going through their [your] mind prior to the alarm sound” and then underwent a structured interview via intercom. Participants were also asked whether they felt fear. Twenty‐second epochs of EEG recording prior to each awakening were then sorted as a function of the presence or absence of fear in the dream. (b) Brain maps showing modulations of delta and gamma power during N2 sleep and delta power during REM sleep when comparing trials with fear to those without fear. Only significant differences at the *p* < .05 level, obtained after correction for multiple comparisons, are shown at the source level (two‐tailed, paired *t*‐tests, TFCE corrected).

Dream sampling during sleep was accomplished using the “serial awakening” method, as described in detail elsewhere (Siclari et al., 2017) (Figure 1a). In brief, participants were awakened several times during the night while sleeping and were asked to describe “*the last thing going through your mind prior to the alarm sound*,” and then underwent a structured interview via intercom. Among other questions, they were asked to name any specific emotion that they experienced, and to report the presence/absence of fear or anxiety. Awakenings were performed at intervals of at least 20 min in N2 sleep or REM sleep using an alarm sound. Participants must have been asleep for a minimum of 10 min and must have been in a stable sleep stage for a minimum of 5 min before any experimental awakening was triggered.

#### 2.2.2 EEG recordings

Recordings were made at the University of Wisconsin (Wisconsin Institute for Sleep and Consciousness) using a 256‐channel hdEEG system (Electrical Geodesics, Inc., Eugene, OR) combined with Alice Sleepware (Philips Respironics, Murrysville, PA). Additional polysomnography channels were used to record and monitor eye movements and submental electromyography during sleep. Sleep scoring was performed over 30 s epochs according to standard criteria (Iber, Ancoli‐Israel, Chesson, & Quan, 2007).

#### 2.2.3 EEG Preprocessing

The EEG signal was sampled at 500 Hz and band‐pass filtered offline between 1 and 50 Hz. The EEG data were high‐pass filtered at 1 Hz instead of lower frequencies as there were sweating artifacts in some of the participants which caused intermittent high‐amplitude (>300 μV) slow frequency oscillatory activity around 0.3 Hz. Noisy channels and epochs containing artifactual activity were visually identified and removed. To remove ocular, muscular, and electrocardiograph artifacts, we performed independent component analysis (ICA) using EEGLAB routines (Delorme, & Makeig, 2004; Jung et al., 2000). The previously removed noisy channels were interpolated using spherical splines (EEGLAB). Finally, EEG data were referenced to the average of all electrodes.

### 2.3 EEG signal analysis

#### 2.3.1 Source localization

The cleaned, filtered and average‐referenced EEG signal corresponding to the 20s before the alarm sound was extracted and analyzed at the source level. We only analyzed the EEG data from those participants who reported at least one dream containing fear and one without fear, within the same recording night and for a given sleep stage (N2 or REM). Source modeling was performed using the GeoSource software (Electrical Geodesics, Inc.). A four‐shell head model based on the Montreal Neurological Institute (MNI) atlas and a standard coregistered set of electrode positions were used to construct the forward model. The source space was restricted to 2,447 dipoles in three‐dimensions that were distributed over 7 × 7 × 7 mm cortical voxels. The inverse matrix was computed using the standardized low‐resolution brain electromagnetic tomography (sLORETA) constraint. A Tikhonov regularization procedure (λ = 10^−1^) was applied to account for the variability in the signal‐to‐noise ratio (Pascual‐Marqui, 2002). We computed spectral power density using the Welch's modified periodogram method (implemented with the *pwelch* function in MATLAB; The Math Works Inc, Natick, MA) in 2 s Hamming windows (eight segments, 50% overlap) to decompose the source signals into frequency bands of interest before taking the norm across dimension to produce a single power value for each dipole. We compared epochs associated with the presence of fear in dreams with those without fear.

### 2.4 Statistical analysis

Statistical analysis was carried out in MATLAB. To compare brain activity between trials with fear and those without, source‐space power was averaged within frequency bands according to literature (Bernardi et al. 2019, Olbrich et al. 2014, Marzano et al. 2011, Corsi‐Cabrera et al. 2000) (Delta: 1‐5 Hz, Theta: 5–8 Hz, Alpha: 8‐12 Hz, Sigma: 12‐16 Hz, Beta: 16‐30 Hz, Gamma: 30‐50 Hz). We then averaged the power values within trials with fear and those trials without fear for each participant and for each frequency band separately. Group level analyses used paired two‐sample *t*‐tests (two‐tailed) between the fear and no fear conditions, performed separately for each frequency band, and thresholded at corrected *p* < .05 using nonparametric threshold‐free cluster enhancement (TFCE; weighing parameters E = 2/3 and H = 2) (Mensen & Khatami, 2013).

### 2.5 Study 2: Modulation of brain responses to aversive stimuli during wake as a function of fear in dreams

#### 2.5.1 Participants

A total of 127 healthy individuals (45 males, age 22.00 ± 3.15 years, 18–37 [mean ± *SD*, range]) participated in Study 2. All participants had no history of neurological or psychiatric disorder. Signed informed consent was obtained from all participants before the experiment, and ethical approval for the study was obtained from the University of Geneva (Switzerland) and University of Liège (Belgium). Among these 127 participants, 13 participants did not report any dream in their dream diary before the experimental session (see below). For the fMRI analyses, we also excluded 25 participants who were presented with emotional (and neutral) words, unlike all other participants who saw emotional pictures. The final group of 89 participants included 58 females, 21.5 ± 2.4 (mean ± *SD*) year‐old. Participants took part in one of three different experiments (Experiment 1: *N* = 28, age 21.36 ± 2.70 years, 16 men, University of Geneva; Experiment 2: *N* = 19, age 22.16 ± 2.48 years, 19 men, University of Geneva, Experiment 3: *N* = 42, age 21.31 ± 2.08, 23 men, University of Liège). All participants filled out the same questionnaires on sleep quality (PSQI, Buysse, et al., 1989), daytime sleepiness (Epworth sleepiness scale, ESS, Johns, 1991), depression (beck depression scale, BDI, Beck, Steer, & Brown, 1996), anxiety (state–trait anxiety inventory trait, STAI‐T, Spielberger, Gorsuch, & Lushene, 1970), and also kept the same sleep and dream diary. Study 2 correspond to a secondary analysis because part of the data was already presented elsewhere (Sterpenich et al., 2014; Sterpenich, Ceravolo, & Schwartz, 2017), yet without exploring any of the dream measurements. Note that the sleep and dream diaries were designed specifically for this analysis and the stimuli of the three different studies correspond to similar visual items (emotional and neutral pictures, see below), eliciting similar changes in emotional arousal and in local brain activation (Figure S1).

#### 2.5.2 Collection and analysis of dream data

**Figure 2:**
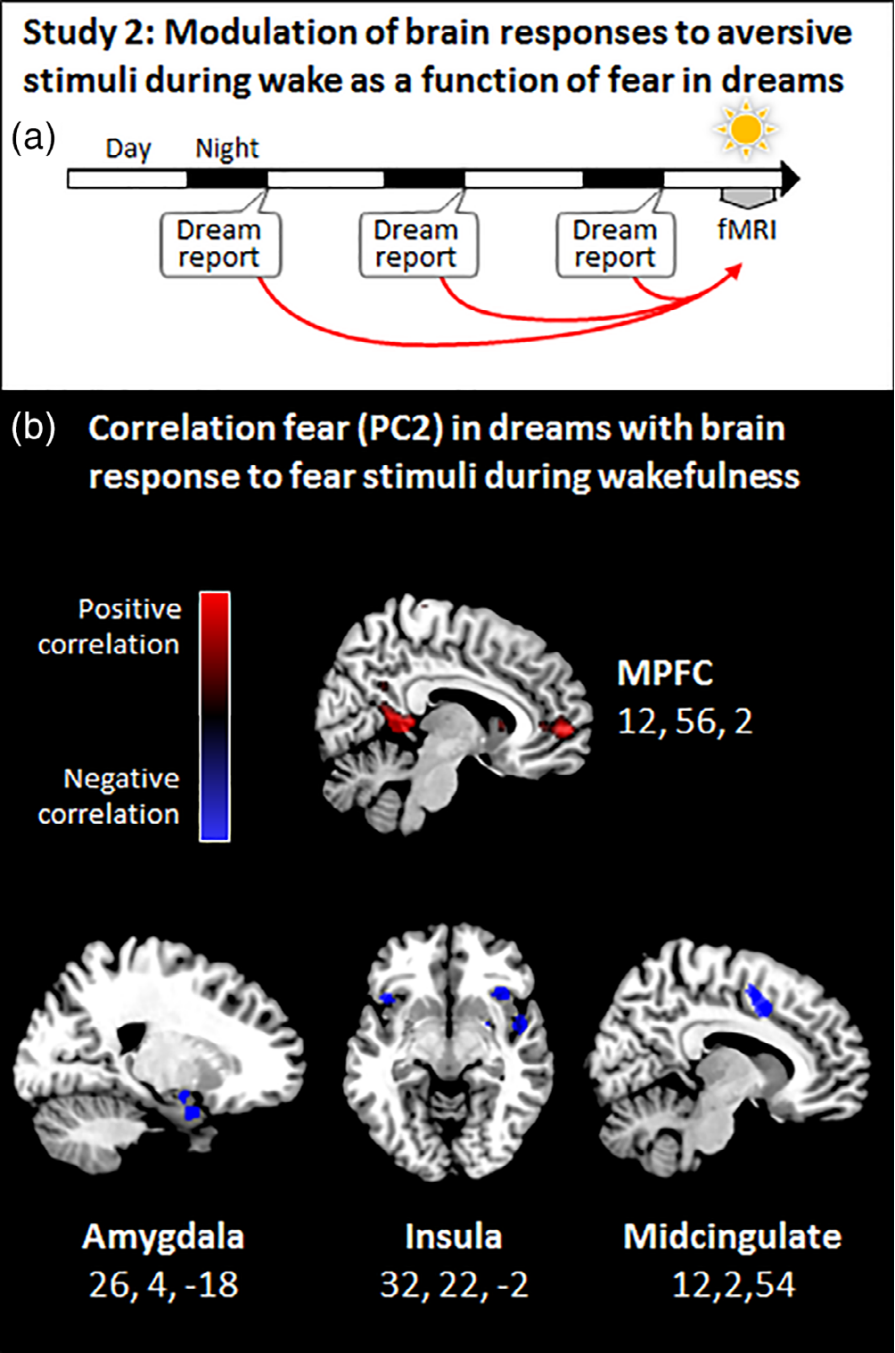
(a) During 1 week, participants filled a dream diary at home, and their responses to aversive stimuli were assessed using fMRI. To test for a link between fear in dreams and brain responses to fear‐eliciting stimuli during wakefulness, the individual propensity to experience fear in dreams (PC2, Table S1) was used as a regressor in a whole‐brain analysis. (b) Responses of the medial prefrontal cortex to aversive stimuli were greater in those individuals who frequently experienced fear in dreams (top panel), while activation of the amygdala, insula, and midcingulate cortex decreased in the same individuals (middle and bottom panels). Significant whole‐brain regression results are displayed on the mean structural image. Results are displayed at *p* < .001 uncorrected on the mean structural image.

During the week preceding the fMRI session, participants were asked to fill out a sleep and dream diary at home (Figure 2a). Every morning, they responded to a mini‐questionnaire about the content of the dreams they may have had during the preceding night, and had the possibility to write down their dreams in more details. Among other questions in the mini‐questionnaire, participants were asked to report the presence/absence of specific emotions in their dreams (anger, disgust, confusion, embarrassment, fear, sadness, joy, and frustration). Note that joyful dreams may be slightly overrepresented because joy was the only positive emotion among the emotions assessed. We computed the percentage of nights with dreams containing specific emotions (for example, three nights with dreams containing fear over a total of five nights with dreams would yield a percentage of fear in dreams of 60%). We entered the percentage for each emotion from each participant (eight values per participant, Table S1) into a principal component analysis (PCA) to (a) identify main affective components (or dimensions) according to variance explained and (b) characterize each participant by component (or factor) scores, which we then used as regressors in an fMRI design matrix. Specifically, here we focused on the individual scores on the second PCA component (PC2) that contrasted basic negative emotions, in particular fear, to nonbasic social negative emotions (such as embarrassment and frustration) (see Schwartz, 2004 for a similar data structure). Because of the imbalanced number of options for negative (*n* = 7) versus positive (*n* = 1) emotions in the original data, we did not investigate the first PCA component (PC1). Indeed, and unsurprisingly, the latter contrasted the only positive emotion to all other (negative) emotions.

### 2.6 Functional MRI session

#### 2.6.1 Emotional tasks

Data from three different fMRI experiments were included in the analysis. Two of these three sets of data have already been reported elsewhere, but none of these former publications concerned emotions in dreams (Sterpenich et al., 2017; Sterpenich, Piguet, et al., 2014). Common to these three experiments was that participants were exposed to aversive and neutral images, and that dream data were collected using the exact same dream diary and under the exact same instructions. These conditions ensured that the combination of the three data sets would yield interpretable results. Below, we briefly describe the task used in each experiment, focusing only on those aspects that are relevant to the purpose of the present work.

In Experiment 1, participants were presented with conditioned (aversive) and unconditioned faces. Stimuli were presented in an intermixed, random order, one at a time (2.5 s each) followed by a varying interval (ISI: 4–5.5 s, mean = 4.75) (see Sterpenich, Piguet, et al., 2014 for more details). In Experiment 2, 60 aversive, 60 funny, and 60 neutral pictures were presented to participants. Each picture was displayed for 3 s, preceded by a fixation cross of 1 s. Participants had a maximum of 2 s to judge the valence of each stimulus on a seven point scale (from −3: very negative, 0: neutral, +3: very funny). In Experiment 3, participants saw 90 faces displaying a negative expression and 90 faces with a neutral expression (see Sterpenich et al., 2017 for more details). Each face was displayed for 3 s, and then participants judged it for emotional valence and arousal. Each trial started with a fixation cross for 1.5‐s duration and ended as soon as participants responded, resulting in a jitter between trials (range 5.03 and 12.1 s). For the analysis of each experiment, we compared activity elicited by the presentation of aversive versus neutral stimuli.

All visual stimuli were presented on a back projection screen inside the scanner bore using an LCD projector, which the participant could comfortably see through a mirror mounted on the head coil. Responses were recorded via an MRI‐compatible response button box (HH‐1 × 4‐CR, Current Designs Inc.).

#### 2.6.2 Pupillary size

During all fMRI sessions, eye movements, and pupil diameter were measured continuously using an MRI‐compatible long‐range infrared eye tracking system (Applied Science Laboratories, Bedford, MA; sampling rate: 60 Hz). Pupil size variation was used as an index of emotional arousal during the tasks (Bradley, Miccoli, Escrig, & Lang, 2008). Pupillary responses were analyzed during epochs of 5 s following the onset of the presentation of pictures. For each epoch, baseline pupil size was estimated as the average pupil measurement during the second preceding the presentation of the picture, and was then subtracted from all values of this epoch. Trials with more than 30% of signal loss were discarded. The pupillary values were z‐scored for each task to take into account the difference in luminance of the different pictures and background. For each trial type (aversive and neutral) and for each task, we analyzed the mean signal value over the 5 s epochs using a *t*‐test (Figure S1A). Data from 50 participants were discarded because of poor quality of the recording or technical problems, and finally data from 77 participants were included in this analysis. The mean pupil diameter value for aversive stimuli was subtracted from that for neutral stimuli for each participant to obtain an individual physiological emotional response to fear‐eliciting stimuli. To test for a potential link with emotions in dreams, we correlated these individual pupil reactivity values with the frequency of fear in dream (PC2).

#### 2.6.3 MRI acquisition

For Experiments 1 and 2, MRI data were acquired on a 3 T whole body MR scanner (Tim Trio, Siemens, Erlangen, Germany) using a 12‐channel head coil. For Experiment 3, data were acquired on a 3 T head‐only magnetic resonance scanner (Allegra, Siemens, Erlangen, Germany). Functional images were acquired with a gradient‐echo EPI sequence with the following parameters. For Experiment 1: repetition time (TR): 2200 ms, echo time (TE): 30 ms, flip angle (FA): 85°, field of view (FOV): 235 mm, 36 transverse slices, voxel size: 1.8 × 1.8 × 3.4 mm. For Experiment 2: TR: 2100 ms, TE: 30 ms, FA: 80°, FOV: 205 mm, 36 transverse slices, voxel size: 3.2 × 3.2 × 3.8 mm. For Experiment 3: TR: 2460 ms, TE: 40 ms, FA: 90°, FOV: 220 mm, 32 transverse slices, voxel size: 3.4 × 3.2 × 3.4 mm. T1‐weighted structural images were also acquired in each participant.

### 2.7 MRI analysis

Functional volumes were analyzed by using statistical parametric mapping 8 (SPM8; http://www.fil.ion.ucl.ac.uk/spm/software/spm8) implemented in Matlab (The MathWorks Inc, Natick, MA). Functional MRI data were corrected for head motion, slice timing, and were spatially normalized to an echo planar imaging template conforming to the Montreal Neurological Institute (MNI) template (voxel size, 3 × 3 × 3 mm). The data were then spatially smoothed with a Gaussian kernel of 8 mm full width at half maximum (FWHM). For each participant, we used a general linear model (GLM) approach to estimate brain responses at every voxel, and computed the main contrast of interest (aversive vs. neutral). For each experiment, any other conditions (e.g., positive items in Experiment 2) were modeled as variable of no interest. Movement parameters estimated during realignment were also added as regressors of no interest. The resulting individual maps of t‐statistics (the contrast images for each individual) were then used in second‐level random‐effects analyses. We used one‐sample *t*‐tests for testing common effects of aversive versus neutral stimuli. For the regression analysis, individual scores on the second PCA component (higher for more fearful basic negative emotion in dreams; see Collection and analysis of dream data section) were used as a regressor at the group level for the contrast aversive versus neutral stimuli. Statistical inferences were corrected for multiple comparisons according to the Gaussian random field theory at *p* < .05 family wise error (FWE) corrected (a) on the entire volume or (b) using correction within predefined anatomical regions (small volume correction, SVC), including the amygdala, insula, and the midcingulate cortex using the toolbox Anatomy (Tzourio‐Mazoyer et al., 2002).

## 3 RESULTS

### 3.1 Study 1: Identifying the neural correlates of fear in dreams

We found significant modulations in the delta (1‐5 Hz) and gamma (30‐50 Hz) ranges, which we interpreted in terms of underlying local neuronal population activity as follows. Increased (respectively decreased) low frequency power in the delta range corresponds to neuronal inhibition (respectively activation) (Pigorini et al., 2015; Tononi & Massimini, 2008), while increased high EEG frequencies in the gamma range reflect increases in neuronal firing (Le Van Quyen et al., 2010; Steriade, Contreras, Amzica, & Timofeev, 1996) and positively correlate with local blood oxygen‐level dependent (BOLD) fluctuations (Murta, Leite, Carmichael, Figueiredo, & Lemieux, 2015).

For NREM, we obtained a total of 79 awakenings in N2 sleep from 12 participants (average per night 6.58 ± 2.13 [mean ± *SD*, range]). Of these awakenings, 57 yielded reports of dream experience (from which 18 without recall of any content), while 22 yielded no report. Fear was present in 14 reports (average per subject 1.16 ± 0.37 [mean ± *SD*, range]) and absent in 25 reports (average per subject 2.08 ± 1.03 [mean ± *SD*, range]). N2 reports with presence of fear (compared to without fear) were associated with decreased delta power in the right insula, and increased gamma power in the bilateral insular cortex (Figure 1b). No significant changes were found for other frequency bands.

For REM sleep, we obtained a total of 32 awakenings from eight participants (average per night 4.00 ± 0.86 [mean ± *SD*, range]). Of these awakenings, 28 yielded reports of dream experience (from which one without recall of any content), whereas four yielded no report. Fear was present in 12 reports (average per subject 1.50 ± 0.50 [mean ± *SD*, range]) and absent in 15 reports (average per subject 1.87 ± 1.05 [mean ± *SD*, range]). REM reports with presence of fear compared to those without fear were associated with decreased delta power in the bilateral insula and midcingulate cortex, thus partly replicating the results from N2 (Figure 1b). No significant changes were found for other frequency bands. Although EEG source reconstruction should be considered with caution, the present data also suggest that experiencing fear in NREM and REM dreams could involve different portions of the insula, with slightly more anterior dorsal insula activity during REM (Figure 1b). Together these results demonstrate that the occurrence of frightening dreams coincided with increased activation of the insular cortex during both NREM and REM sleep, and of the midcingulate cortex during REM sleep.

### 3.2 Study 2: Linking awake brain responses to aversive stimuli and fear in dreams

After we established that fear in dreams implicated the insula and midcingulate cortex known to contribute to the processing of aversive stimuli during wakefulness (Study 1), we asked whether frequently experiencing fear in dreams related to individual autonomic and neural sensitivity to fear during wakefulness. All participants filled the same sleep and dream diary over 7.38 (± 4.90; mean ± *SD*) nights with the same instructions before an MRI session (with a minimum of three nights). From the sleep diary, participants reported a mean sleep duration of 7.93 (± 0.76 *SD*) hours per night and a sleep quality of 7.02 (± 0.97 *SD*; on a 10‐point scale from 0—very bad sleep to 10—very good sleep quality). From the dream diary, an average of 5.47 nights (± 3.63 *SD*) were associated with the feeling of having dreamt, and participants answered specific questions related to the content of their dreams after 3.75 (± 2.66 *SD*) nights. The first component (PC1; 20.39% of the variance) distinguished between negative and positive (here “joy”) emotions, thus representing emotional valence. The second component (PC2; 16.40% of the variance) contrasted emotions related to basic negative emotions (fear, anger, sadness, and disgust), with fear having the strongest contribution, and those related to nonbasic, social negative emotions (i.e., embarrassment, confusion, frustration; see Schwartz, 2004 for a similar finding). The predominance of fear reported in dreams provides a first confirmation of our initial hypothesis about the expression of this emotion during sleep. Accordingly, we shall call this second “fear component” or PC2 in the remainder of the manuscript. The individual PCA scores (or loadings) for the fear component did not correlate with sleep quality (PSQI, R^2^ < 0.001, *p* = .51), sleep duration (sleep diary, R^2^ = 0.005, *p* = .44), sleepiness (ESS, R^2^ = 0.02, *p* = .12), depression (BDI, R^2^ = 0.008, *p* = 0.34), anxiety (STAI‐T, R^2^ = 0.001, *p* = .77), or frequency of dreaming (percentage of nights with dreams, R^2^ = 0.003, *p* = .54). This pattern of results supports that the PC2 might represent an individual affective measure that is largely independent of other sleep‐related variables and waking affective dimensions.

Study 2 aimed at establishing whether neurophysiological responses to fear‐eliciting emotions during wakefulness correlated with fear in dreams. We first analyzed changes in pupillary size recorded during the presentation of aversive (vs. neutral; see Methods; Figure S1a) stimuli as an index of emotional arousal response (Bradley et al., 2008), and showed that this index correlated with the individual PC2 (Spearman's rho = 0.25, *p* = .034). Specifically, higher fear load in dreams was associated with lower mydriasis in response to fear‐eliciting stimuli while awake. We next tested whether we could observe a similar relationship between emotions in dreams and brain responses collected during the presentation of the same aversive stimuli (vs. neutral). Using fMRI, we first confirmed that fear‐eliciting stimuli activated a set of expected brain regions, including the amygdala, the insula, and occipital regions (Table S2, Figure S1b). Then, we used the PC2 as a regressor in a whole‐brain regression analysis (for the contrast aversive vs. neutral stimuli, see Methods). This analysis revealed that participants with a high propensity to experience fear in dreams had increased activation of the medial prefrontal cortex (Figure 2b, Table S3) when facing aversive stimuli while awake. Conversely, and as predicted by theoretical models (see Introduction), the same analysis yielded negative correlations with activity in the right amygdala, right insula, and midcingulate cortex (Figure 2b). Moreover, the activation of the insula (for negative vs. neutral items modulated by PC2) also correlated with larger pupillary size during the presentation of aversive versus neutral items (R^2^ = 0.1, p = .005). We also performed a regression analysis by adding the change in pupillary size (negative vs. neutral items) as a covariate in the main contrast aversive versus neutral pictures and reported a significant recruitment of the insula region bilaterally (Figure S2) among other regions. Finally, we performed the same regression between BOLD for aversive versus neutral stimuli during wakefulness and individual PC1 scores. We observed an activation of the midcingulate cortex (Figure S3) and no activation for the reverse contrast. Note that sleep parameters (sleep quality and duration) did not show any significant activation when used as covariates for the contrast of aversive versus neutral pictures.

## 4 DISCUSSION

Here we investigated the neural correlates of fear in dreams and their relation to brain responses to threatening stimuli during wakefulness. In Study 1, we found that experiencing fear (vs. no fear) in dreams was associated with the activation of the insula and midcingulate cortex (the latter during REM dreams; Figure 1b), which were both also activated when experiencing fear during wakefulness (Study 2; Figure S1b, Table S2), as also classically reported in previous research (Alves et al., 2013; Casanova et al., 2016; Pereira et al., 2010). We recently reported that specific dream contents—such as faces, places, movement, speech, and thoughts—engage similar cortical networks as during wakefulness (Perogamvros et al., 2017; Siclari et al., 2017). Here we show, for the first time to our knowledge, that fear‐related experiences activated the insula during both dreaming and awake consciousness. The consistency of the present results across brain states and their correspondence with classical work on brain structures involved in fear are encouraging. Importantly, hdEEG (especially with 256 electrodes as in our study) can have sufficient accuracy in source localization (e.g., associating dreams containing faces with activation of the fusiform face area; Siclari et al., 2017), even for the detection of signal originating from deep cerebral structures (Seeber et al., 2019). However, combined EEG/fMRI studies and a higher number of observations per condition (i.e., presence and absence of fear) are certainly needed to further elucidate the exact contribution of subcortical structures, such as the amygdala, especially during REM sleep (Corsi‐Cabrera et al., 2016; De Gennaro et al., 2011; Maquet et al., 1996).

The insula, particularly its anterior part, may contribute to social–emotional experience and associated visceral states, possibly giving rise to conscious feelings (Chang, Yarkoni, Khaw, & Sanfey, 2013; Critchley, Wiens, Rotshtein, Ohman, & Dolan, 2004), and participates in the emotional response to distressing cognitive or interoceptive signals (Reiman et al., 1997). Insula activation during dreaming could thus reflect the integration of internally generated sensory, affective, and bodily information culminating in a subjective feeling of danger, as we further discuss below. During REM sleep, we also found an activation of the midcingulate cortex, a region known to be critically involved in behavioral/motor responses to dangers (Pereira et al., 2010). Because REM sleep is characterized by activation across sensory and motor cortices (Schwartz & Maquet, 2002), and by muscle atonia that prevents the overt expression of motor behaviors, this sleep stage could provide a well‐suited physiological condition for the (re)activation of threatening situations with associated emotional and motor reactions.

Based on the results from Study 1, which suggested an anatomo‐functional correspondence between fear in dreams and wakefulness, we then asked whether frequently experiencing fear in dreams (PC2) might relate to the individual's neurophysiological sensitivity to fear during wakefulness. In Study 2, we analyzed awake fMRI response to aversive stimuli as a function of whether participants reported a high incidence of fear in their dreams. Here we thus considered fear in dreams as an individual trait, which we determined based on the analysis of a large data set of dreams. Of note, this measure of PC2 did not correlate with depression, anxiety, sleep quality, and the frequency of dream recall (all *p* > .05), which supports the specificity of fear in dreams with respect to other classical dimensions of mood or sleep/dreams. We found decreased activity in the insula and amygdala, while activity of the mPFC cortex was increased (Figure 2b). Along with the insula, both the amygdala and the cingulate cortex have been associated with fear and the perception of negative emotions during wakefulness (Phan et al., 2002), as we also demonstrated (Figure S1b, Table S2). Conversely, the mPFC is believed to regulate the response to threatening stimuli by modulating the activity of the amygdala (Phelps et al., 2004; Quirk et al., 2003). Specifically, the mPFC exerts an inhibitory control on fear expression by decreasing amygdala output and has been associated with extinction learning (i.e., when a neutral conditioned stimulus that previously predicted an aversive unconditioned stimulus no longer does so, and conditioned response subsequently decreases) (Herry et al., 2010; Kalisch et al., 2006). Consistent with the proposal that dreaming may serve an emotion regulation function (Cartwright et al., 2006; Hartmann, 1996; Kramer, 1991; Perogamvros, Dang‐Vu, Desseilles, & Schwartz, 2013), participants who frequently (but not excessively, see below) experienced frightening dreams showed a stronger inhibition of the amygdala, potentially mediated by the mPFC. This interpretation is further supported by the pupillary results showing that participants who frequently reported fear in their dreams had reduced autonomic responses to aversive stimuli during wakefulness, suggesting a better ability to regulate defensive and alerting reactions to threatening signals in those individuals.

In the domain of sleep research (irrespective of dreaming), REM sleep was found to play a role in emotional memory consolidation (Goldstein & Walker, 2014; Nishida, Pearsall, Buckner, & Walker, 2009; Sterpenich et al., 2014), including fear memory consolidation (Pace‐Schott, Germain, & Milad, 2015) and successful fear/safety recall (Menz, Rihm, & Buchel, 2016), while both NREM (Hauner, Howard, Zelano, & Gottfried, 2013; He et al., 2015) and REM sleep stages (Menz et al., 2016; Pace‐Schott et al., 2015) have been found to promote the retention and generalization of extinction learning. In addition, it was proposed that specifically REM sleep contributes to the attenuation of the emotional tone of waking‐life memories (Vallat, Chatard, Blagrove, & Ruby, 2017; Walker & van der Helm, 2009). Importantly, total sleep deprivation may cause a reduction of mPFC control over the limbic system, resulting in an accentuation of emotional responses to negative stimuli (Yoo et al., 2007) and an impairment of extinction recall (Straus, Acheson, Risbrough, & Drummond, 2017). We have previously suggested that these findings from sleep studies may putatively extend to or even depend on concomitant dreaming (Perogamvros et al., 2013; Perogamvros & Schwartz, 2012). In particular, the exposure to feared stimuli (objects, situations, thoughts, memories, and physical sensations) in a totally safe context during dreaming would thus resemble desensitization therapy (Levin & Nielsen, 2007). Besides, several studies have demonstrated that dreaming of negative waking‐life experiences (e.g., divorce) relates to the resolution of previous emotional conflicts and a reduction of next‐day negative mood (Cartwright et al., 2006; Cartwright, Lloyd, Knight, & Trenholme, 1984). While this was not the objective of the present work, we would like to emphasize that the present data do not allow us to make any inference about whether one occurrence of one specific emotion in a dream influences the emotional state or responsiveness on the following day. Moreover, correlational results cannot be interpreted in a unidirectional and/or causal way. Indeed, we could also suggest that fear suppression during wakefulness may lead to increased excitability of fear‐related experiences during sleep, namely in a condition where monitoring (emotion regulation) from the prefrontal cortex is reduced (Braun et al., 1997; Maquet et al., 1996).

Contrasting with this potentially beneficial role of negative but benign dreams, recurrent nightmares, such as those observed in PTSD patients, could represent a failure of the fear extinction function of dreaming (Nielsen, 2017; Nielsen & Levin, 2007). Thus, nightmare patients would be more prone to emotional dysregulation, as suggested by one recent study reporting decreased mPFC activity during the viewing of negative pictures in these patients (Marquis et al., 2016). Furthermore, exerting ineffective emotional regulation strategies (e.g., fear suppression) and elevated anxiety during wakefulness may lead to increased excitability of negatively‐loaded memories at sleep‐onset or even during sleep (Malinowski, 2017; Schmidt & Gendolla, 2008; Sikka, Pesonen, & Revonsuo, 2018; Sikka, Revonsuo, Noreika, & Valli, 2019), namely in conditions where monitoring from the prefrontal cortex is reduced (Braun et al., 1997; Maquet et al., 1996). Such disruption in the regulation of emotions during wakefulness and sleep has been proposed as a major contributing factor to insomnia (Wassing et al., 2016).

Here, we experimentally show that, beyond sleeping, experiencing negative emotions specifically during dreaming is associated with better‐adapted emotional responses during waking life. Study 2 combined data from three different experiments testing for brain responses to aversive stimuli (vs. neutral). This allowed us to include a very large set of participants, which is needed to exploit interindividual differences, as we do here. In all three experiments, dream reports were collected using the exact same instructions and same questionnaires. While we confirmed consistent fMRI and pupillary response results across the three experiments for the effects of aversive vs. neutral emotions, Study 2 yielded significant results in brain regions, for which we had strong theory‐driven a priori. We therefore suggest that what may be perceived as a potential limitation (i.e., combining data from three experiments) may actually offer a better generalizability of the present findings to diverse waking threatening situations. Indeed, collecting the same behavioral variable (i.e. emotions in dreams) across studies prevents that any reported correlation between emotions in dreams and fMRI signal from the ROIs arises from between‐study differences in fMRI responses.

Taken together, across two complementary studies, we show opposing neural effects of fear experience in dreams and during wakefulness. These results show that individuals who reported a high prevalence of fear‐related emotions in their dreams had stronger fear inhibition during wakefulness. Critically, associated neural decreases implicated the insula, which was strongly activated during fearful dreams in Study 1, thus further supporting reciprocal links between sleep and wake emotional functioning. While both studies use distinct conceptual and methodological approaches, they offer necessary complementary information about affective processes in the brain during dreaming and wakefulness, including their potential links. These results also support recent theoretical claims that dreaming (beyond sleep) benefits emotion regulation processes, by achieving a form of overnight affective simulation or recalibration (e.g., through extinction learning and generalization), which would foster adapted emotional responses to dangerous real‐life events (Nielsen & Levin, 2007; Perogamvros & Schwartz, 2012; Revonsuo, 2000). Studying the role of positive emotions (e.g., positive social interactions) in dreams (especially in NREM dreams; McNamara, McLaren, Smith, Brown, & Stickgold, 2005) and their potential links with emotional brain responses during wakefulness may be needed to further corroborate or refine existing theoretical models. Finally, based on our results, we would like to suggest that future studies should address how sleep and dreaming may influence exposure and extinction‐based therapies for affective and anxiety disorders (Pace‐Schott et al., 2018).

## CONFLICT OF INTEREST

The authors do not have any conflicts of interest to disclose.

## Supporting information


**Appendix** **S1**: SUPPLEMENTAL INFORMATION

## Data Availability

The data that support the findings of this study are available on request from the corresponding author. The data are not publicly available due to privacy or ethical restrictions.
