# Supplementary material for: Fear in dreams and in wakefulness: Evidence for day/night affective homeostasis
Source: Hum Brain Mapp. 2019 Oct 30;41(3):840–50. doi: 10.1002/hbm.24843 (PMC7267911; doi:10.1002/hbm.24843)
Supplement: Supplementary file 1 — Appendix S1: SUPPLEMENTAL INFORMATION [file HBM-41-840-s001.docx]

**SUPPLEMENTAL INFORMATION**

**Table S1:** PCA analysis of the emotions in dreams.

| **Type of emotion** | **Percentage of emotion in dreams** | **PCA component 1 (correlation coefficient)** | **PCA component 2 (correlation coefficient)** |
| --- | --- | --- | --- |
| Fear | 19.42 ± 27.38% | -0.40 | 0.36 |
| Anger | 15.63 ± 23.11% | -0.65 | 0.16 |
| Sadness | 9.65 ± 20.10% | -0.41 | 0.07 |
| Disgust | 5.15 ± 13.82% | -0.62 | 0.04 |
| Frustration | 13.21 ± 21.16% | -0.62 | -0.33 |
| Confusion | 18.05 ± 25.44% | -0.35 | -0.53 |
| Embarrassment | 8.31 ± 18.59% | -0.08 | -0.59 |
| Joy | 34.84 ± 32.41% | 0.22 | -0.64 |

**Table S2:** Functional MRI responses to aversive (vs. neutral) stimuli across the three fMRI experiments. Results are corrected for multiple comparisons using family-wise error correction at p<0.05 (FWE-corr) i) on the entire volume or ii) on predefined anatomical regions of interest (marked as ^+^).

| **Name** | **MNI coordinates** | **Z-score** | **P _FWE-corr_** |
| --- | --- | --- | --- |
| ***Aversive > Neutral*** | | | |
| Amygdala | 20, -4, -16 | 4.51 | <0.001^+^ |
| Anterior insula | -36, 26, -6 | 6.24 | <0.001 |
|  | 32, 16, -18 | 5.20 | 0.003 |
| Inferior frontal gyrus | -32, 14, -26 | 5.87 | <0.001 |
|  | 44,32,-4 | 5.64 | <0.001 |
| Motor cortex | -42, 0, 46 | 4.95 | 0.010 |
| Anterior middle temporal gyrus | 50, -2, -24 | 6.74 | <0.001 |
| Posterior middle temporal gyrus | -54, -58, 10 | 6.16 | <0.001 |
|  | 52, -56, 14 | 6.06 | <0.001 |
| Thalamus | 0,-16,-4 | 6.45 | <0.001 |
| Superior temporal sulcus | 50, -18, 10 | 5.75 | <0.001 |
| Calcarine/lingual gyrus | -10, -78, 8 | 4.87 | 0.015 |
|  | 20, -60, 4 | 5.19 | 0.003 |
| Cuneus | 14, -100, 6 | 4.98 | 0.009 |
|  | -28, -86, -6 | 4.90 | 0.013 |
| Midcingulate cortex | 10, 18, 42 | 4.43 | 0.002^+^ |
| Superior frontal sulcus | 42, 12, 24 | 5.04 | 0.007 |
| ***Aversive < Neutral*** | | | |
| Medial PFC | 10, 44, -8 | 4.77 | 0.009 |
| Motor cortex | -52, -16, 50 | 4.91 | 0.013 |
| Angular gyrus | 50, -62, 46 | 4.99 | 0.009 |

**Table S3:** Whole-brain correlation between fMRI activity elicited by aversive (vs. neutral) stimuli during wakefulness and basic (fear) emotions in dreams (as captured by the score on the second PCA component). Results are corrected for multiple comparisons using family-wise error correction at p<0.05 (FWE-corr) i) on the entire volume or ii) on predefined anatomical regions of interest (marked as ^ᵻ^).

| **Name** | **MNI coordinates** | **Z-score** | **P _FWE-corr_** |
| --- | --- | --- | --- |
| ***Positive correlations*** | | | |
| Medial PFC | 12,56,2 | 5.59 | 0.004 |
| ***Negative correlations*** | | | |
| Amygdala | 26, 4, -18 | 3.48 | 0.008 ^ᵻ^ |
| Amygdala/striatum | 22,0,-12 | 3.48 | 0.006 ^ᵻ^ |
| Anterior insula | -30, 26, 4  32, 22, -2 | 3.33  3.65 | 0.058 ^ᵻ^  0.019 ^ᵻ^ |
| Midcingulate cortex | 12, 2, 54 | 4.68 | 0.034 |

**
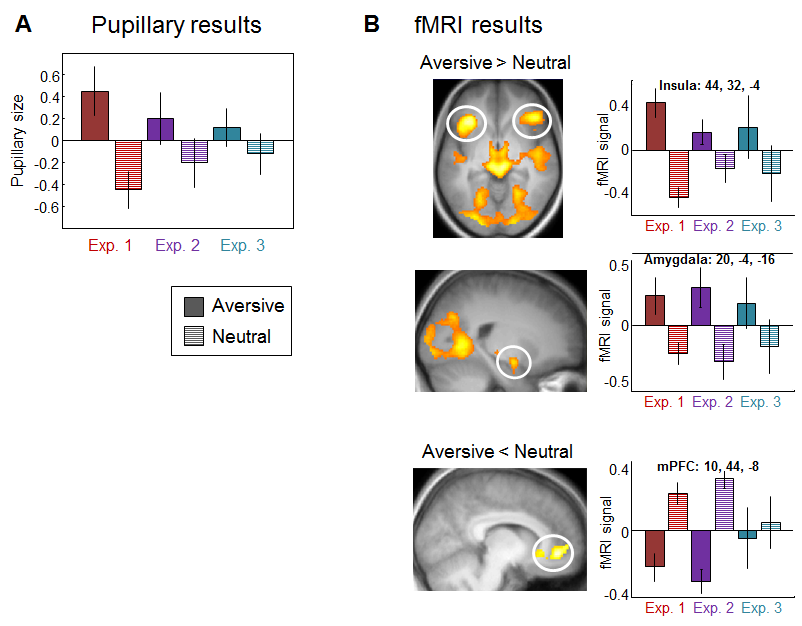
**

**Figure S1. A.** Pupil diameter change in response to aversive and neutral stimuli, showing a significant difference between aversive (plain color) and neutral (hatched) conditions for each experiment (Exp. 1: t=4.79, p<0.001; Exp. 2: t=5.15, p<0.001; Exp. 3: t=3.49, P=0.002).

**B.** Regional changes in fMRI signal in response to aversive vs. neutral stimuli across the three fMRI experiments. Right panels show the parameters estimates for each experiment separately for aversive and neutral stimuli. For display purposes, whole-brain results are displayed on the mean structural image at P=0.001 uncorrected.


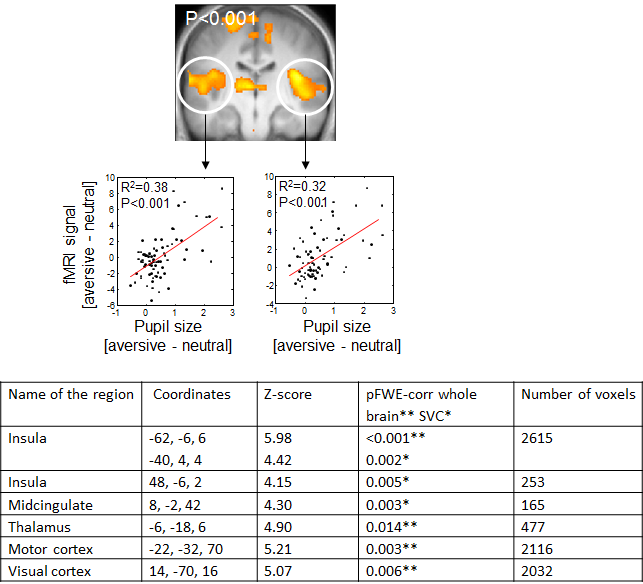


**Figure S2:** Brain activations in response to aversive (vs. neutral) stimuli positively correlated with increased pupillary response to the same aversive (vs. neutral) stimuli


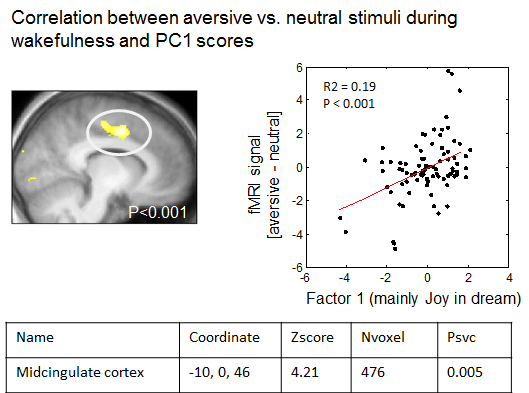


**Figure S3**: correlation between aversive vs. neutral stimuli during wakefulness and PC1 scores. On the left panel, brain activation is displayed on the mean structural image. On the right panel, extracted parameter estimates of the Midcingulate cortex are correlated with the PC1 (referring mainly to the amount of joy in dream)
